# Nicotinamide mononucleotide restores oxidative stress‐related apoptosis of oocyte exposed to benzyl butyl phthalate in mice

**DOI:** 10.1111/cpr.13419

**Published:** 2023-02-09

**Authors:** Yi Jiang, Di Wang, Cheng Zhang, Yangyang Jiao, Yanan Pu, Rong Cheng, Chunyu Li, Yan Chen

**Affiliations:** ^1^ Outpatient & Emergency Management Department The First Affiliated Hospital of Nanjing Medical University Nanjing China; ^2^ Emergency Management Department, School of Health Policy & Management Nanjing Medical University Nanjing China; ^3^ Research Institute of Health Jiangsu Nanjing Medical University Nanjing China

## Abstract

Benzyl butyl phthalate (BBP) is a chemical softener and plasticizer commonly used in toys, food packaging, wallpaper, detergents and shampoos. The estrogenic actions of BBP have detrimental effects on humans and animals. In this study, the specific influence of BBP on mouse oocyte maturation was investigated using in vivo and in vitro models. The experiment first verified that BBP exposure significantly affected the rate of oocyte exclusion of the first polar body, although it did not affect germinal vesicle breakdown (GVBD) through in vitro oocyte culture system. Results of in vitro fertilization show that BBP exposure affects blastocyst rate. Subsequently, the results obtained by immunofluorescence staining technology showed that oocyte spindle organization, chromosomal arrangement and the distribution of cortical actin were disrupted by BBP exposure, and led to the failure of oocyte meiotic maturation and the subsequent early embryo development. Singe‐cell transcriptome analysis found that BBP exposure altered the expression levels of 588 genes, most associated with mitochondria‐related oxidative stress. Further analysis demonstrated that the detrimental effects of BBP involved the disruption of mitochondrial function and oxidative stress‐induced early apoptosis. Nicotinamide mononucleotide (NMN) supplementation reduced the adverse effects of BBP. Collectively, these findings revealed a mechanism of BBP‐induced toxicity on female reproduction and showed that NMN provides an effective treatment for BBP actions.

## Graphical abstract

Benzyl butyl phthalate (BBP) exposure induced mitochondrial dysfunction, causing reactive oxygen species accumulation and thereby leading to oxidative stress‐mediated DNA damage and apoptosis, which ultimately resulted in meiotic defects in oocytes and fertilization failure in mice. Moreover, this study found that nicotinamide mononucleotide administration reduced BBP toxicity and improved oocyte quality.

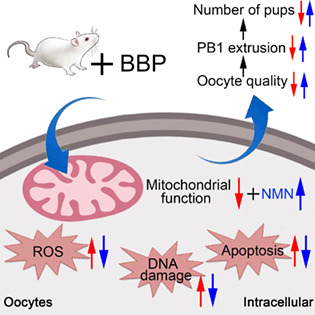

## 1 INTRODUCTION

Benzyl butyl phthalate (BBP) is a widely used chemical softener and plasticizer that is regarded as an environmental pollutant because of its estrogenic activity and pathogenicity.^1^, ^2^ Humans have ubiquitous exposure to BBP and its metabolites through phthalate‐containing medications and products, and the slow release of these phthalates from such products elevates the risk to human health.^3^ BBP is a type of phthalate ester, chemicals which are closely associated with autism.^4^, ^5^ Exposure to BBP has been reported to cause features of endometriosis.^6^, ^7^ Furthermore, animal experiments have demonstrated that exposure to phthalate and its metabolites can adversely damage the reproductive system and oestrogen‐sensitive tissues by affecting circulating hormones.^3^ It was reported that chronic exposure to phthalates changed the biogenesis of ovarian miRNAs in mice, leading to upregulated expression of hormone synthesis‐related miRNAs, thus inducing reproductive dysfunction.^8^ Dietary exposure to BBP generated significant systemic and reproductive toxicity in rats.^9^ However, the specific influence and mechanisms of BBP actions on oocyte maturation and early embryo development remain unknown.

As an established cofactor of diverse physiological enzymes, nicotinamide adenine dinucleotide (NAD+) is required for critical redox reactions in organisms.^10^ Nicotinamide mononucleotide (NMN) is the synthetic precursor of NAD+, converted to NAD+ via adenylyl transferases, and it plays an indispensable role in the maintenance of cellular NAD+ content.^11^, ^12^ Promoting NAD+ biosynthesis by the administration of NMN provides an efficient approach for treating many pathological conditions.^12^ Both long‐term and short‐term NMN intake have significant therapeutic effects on many diseases, such as metabolic complications.^13^, ^14^, ^15^ Moreover, NMN can improve neuronal and muscular function, and extend lifespan.^16^ It has been shown that NMN supplementation can reverse detrimental effects of aging during in vitro and in vivo mouse oocytes maturation, fertilization, and the subsequent early embryo development.^17^ Many studies have shown that increasing NAD+ synthesis by NMN supplementation can alleviate aging‐induced organic dysfunction.^12^, ^13^, ^14^ Overall, NMN supplementation has been effective in maintaining the capacity of oocyte maturation and early embryo development in mice.

In this study, the toxic effects and underlying mechanisms of BBP actions on oocytes maturation in mice were investigated. Our study revealed that BBP exposure disrupted the spindle assembly, kinetochore–microtubule (K‐M) attachment, and oocyte euploidy, and caused polar body extrusion and early embryo development failure. The administration of NMN significantly reduced the level of early apoptosis caused by reactive oxygen species (ROS) production and excessive oxidative stress.

## 2 MATERIALS AND METHODS

### 2.1 Antibodies

Mouse anti‐α‐tubulin‐FITC monoclonal antibody, mouse anti‐acetyl‐α‐tubulin (Lys 40) monoclonal antibody, and phalloidin‐TRITC were obtained from Sigma Company (St. Louis, Missouri). Rabbit anti‐human γH2AX polyclonal antibody and rabbit anti‐glyceraldehyde‐3‐phosphate dehydrogenase (GAPDH) monoclonal antibody were obtained from Cell Signalling Technology (Danvers, Massachusetts). Human anti‐centromere antibody was obtained from Antibodies Incorporated (Davis, California).

### 2.2 Oocytes collection and culture

Animal Care and Use Committee of The First Affiliated Hospital of Nanjing Medical University permitted all experiments and all processes were carried out according to the Animal Research guidelines. The mice used in this experiment were 5–6 weeks old female ICR mice. Mice were placed in a room with proper temperature and light. Oocytes were obtained from each group ovaries and washed briefly with clean medium 48 h after injection of pregnant mare serum gonadotrophin (PMSG). Finally, the oocytes were cultured using M16 medium covered with paraffin oil in a specific incubator for further experiments. If the oocytes are taken from in vivo, PMSG was injected intraperitoneally and then human chorionic gonadotrophin (hCG) was injected 48 h later. oocytes are expelled from the follicle and obtained from each group ampullaes 12–14 h after the injection of hCG.

### 2.3 In vivo fertilization and embryo culture

Female mice were first injected with PMSG and were injected with hCG after 48 h. Then, female mice in each group and healthy male mice were mated, and 12 h after mating the fertilized eggs were obtained from the female mice. The zygotes were then cultured in KSOM‐covered mineral oil in specific incubator to obtain embryos.

### 2.4 BBP and NMN treatment

For the in vivo experiment, mice were divided into three groups randomly and were oral gavage with 0, 0.5, or 1.5 mg/kg BW/day of BBP (AccuStandard, ALR‐082 N) which dissolve in corn oil, respectively. Based on the effects of adding different concentrations of BBP exposure on oocytes maturation in vivo, we applied 1.5 mg/kg BW/day of BBP as a finally operation concentration. BBP‐exposed mice were administrated continuously with BBP for 8 days, and hormones were injected on Days 8 and 10 to promote superovulation. For rescue experiment, mice were treated with NMN (200 mg/kg BW/day, dissolved in phosphate buffered saline (PBS)) by intraperitoneal injected along with the administration of BBP.

### 2.5 Immunofluorescence staining and confocal microscopy

For denuded oocytes, at room temperature, 4% paraformaldehyde was applied for 30 min, then 0.5% Triton X‐100 was applied for 20 min. At room temperature, oocytes were incubated in 1% Bachelor of science in Agriculture blocking buffer for 1 h before incubation with anti‐a‐tubulin‐FITC antibody (1:200), anti‐centromere antibody (1:200) or phalloidin‐TRITC (1:100), followed by incubation with Goat anti‐human immunoglobulin G (H + L), Alexa Fluor 555 for 1 h. After counterstained for 10 min with 10 μg/mL propidium iodide or Hoechst 33342, we mounted these oocytes on glass slides and used confocal microscopes (LSM 900, Carl Zeiss) to capture images. For mitochondrial distribution, MitoTracker Red CMXRos (ThermoFisher; 500 nM) was added to M16 medium at 37°C for 30 min to stain oocytes. Additionally, the mitochondrial membrane potential was evaluated by growing oocytes in M16 medium with MitoProbe JC‐1 (ThermoFisher) (2 μM) in the same way.

In order to measure fluorescence intensity, oocytes in two groups were immunostained similarly and confocal microscope settings were identical. A region of interest was defined with the software called ImageJ (NIH, Bethesda, Maryland), and fluorescence intensities per unit area were determined. Comparisons between control and HS‐treated groups were based on the average intensities of all measurements.

### 2.6 ROS detection and Annexin‐V Staining

To detect the level of ROS in oocytes from each group, we used dichlorofluorescein (DCFH) diacetate (DCFHDA) kitfor living cell staining. Oocytes obtained from each group were incubated in M16 medium containing 10 pM DCFHDA (1:800) in an incubator for 30 min. Subsequently, the oocytes were briefly washed with dulbecco's phosphate‐buffered saline (DPBS) and placed onto the glass slides. The ROS fluorescent signals in oocytes were finally picked by scanning install (Zeiss LSM 700 META). To detect the level of early apoptosis in oocytes from each group, we performed Annexin‐V staining. After brief wash with PBS, oocytes were stained with 90 pl buffer containing 10 pl Annexin‐V‐FITC away from light for 30 min. Oocytes were subsequently placed to the glass slides after brief wash with DPBS. The fluorescent signals of Annexin‐V in oocytes were finally examined by scanning install (Zeiss LSM700 META). Experiments were carried out at least three replicates.

### 2.7 Chromosome spread

Fifteen MII oocytes from each group were treated with 1% sodium citrate for 10 min and immediately transferred onto a glass slide. After Hoechst 33342 staining, the chromosomes were detected by confocal microscopy (Zeiss LSM 700 META). Experiments were carried out at least three replicates.

### 2.8 Western blot analysis

A total of 150 germinal vesicle (GV) oocytes were collected from each group and lysed in sodium dodecyl sulfate sample buffer. The samples were immediately boiled at 100°C for 10 min. Boiled samples were put into sodium dodecyl sulfate polyAcrylamide gel electrophoresis for electrophoresis. Then, proteins were transferred onto a polyvinylidene fluoride membrane through transmembrane (Millipore, USA). Subsequently, the protein‐containing membranes were blocked with Tris‐buffered saline containing 5% non‐fat milk and 0.1% Tween 20 (TBST) for 1 h at room temperature. We then washed the membrane transiently with TBST and incubated them with certain primary antibodies at 4°C for a whole night. After washing for five times (5 min/time) in TBST, the membranes were incubated with specific secondary antibodies for 1 h at room temperature. We then washed the membrane transiently with TBST and the membrane was exposed to an enhanced chemiluminescence reagent (from EMD Millipore, Billerica, Massachusetts). Finally, all bands were visualized by Tanon‐3900. Experiments were carried out at least three replicates.

### 2.9 RNA‐seq (transcriptome sequencing)

To illustrate all the transcribed mRNA forms in control oocytes and BBP‐treated oocytes respectively, we used the lllumina Hiseq sequencing platform‐based transcriptome sequencing technology. Ten MII oocytes were contained in each sample. Agilent bioanalyzer 2100 was used to determine RNA quality and NEB Next Ultra RNA library was used to prepare our transcriptome library. The Illumina Hi Seq platform was used to sequence and analyse the biological information after mixing libraries based on the set concentration and volume. Next, Bcl2fastq (v2.17.1.1) was used to analyse the obtained data for base calling and primary quality. We used gene ontology (GO) analysis, Kyoto Encyclopedia of Genes and Genomes (KEGG) analysis to compare our pathway enrichment analysis to the transcriptome background.

### 2.10 Quantitative real‐time PCR

Fifty GV oocytes were collected from each group. RNA contents in oocytes from each group were extracted, and the obtained RNA was reverse transcription to cDNA. PCR reaction system was constituted with 1.2 μL certain primer, 4.8 μL hyperpure water, 10 μL Advanced SYBR Green PCR Master Mix, and 4 μL cDNA sample. The specific genes expression levels were determined by RT‐PCR. RT‐PCR was completed by using a One Step SYBR Prime Script RT PCR Kit (Ta Ka Ra Bio., Inc., Tokyo, Japan). Experiments were carried out at least three replicates. The primers were listed as follows:

Crisp1 (F:TGTTGGCAATTATCAAGGAAGGC/R:TGGTCCTGGCTACGAGTACA).

Scd3 (F: TCCCCTACGACTACTCTGCC /R: ACATGGGTGCTTCTTTTCGGT).

Kif18b (F: GGAACCGACGAGAGGTGTTG/R: TCTTCTTGGGGCCATTGTGG).

Atp6v1c2 (F:CGCGTGTCTTCTACAGCTCA/R:GAGGCCAACAAGGGAATCCA).

Gapdh (F: CCCTTAAGAGGGATGCTGCC/R: TACGGCCAAATCCGTTCACA).

### 2.11 Statistical analysis

Observations were represented as mean ± SEM, which at least three repeated trials, and oocyte counts were cited in parentheses as (*n*). As part of the analysis, GraphPad Prism 7 statistical software was used to carry out a paired samples *t*‐test. The study accepted *p* < 0.05 for significance.

## 3 RESULTS

### 3.1 BBP exposure leads to a decrease in the number of oocyte and antral follicle, as well as the developmental potential of mouse oocyte

**FIGURE 1:**
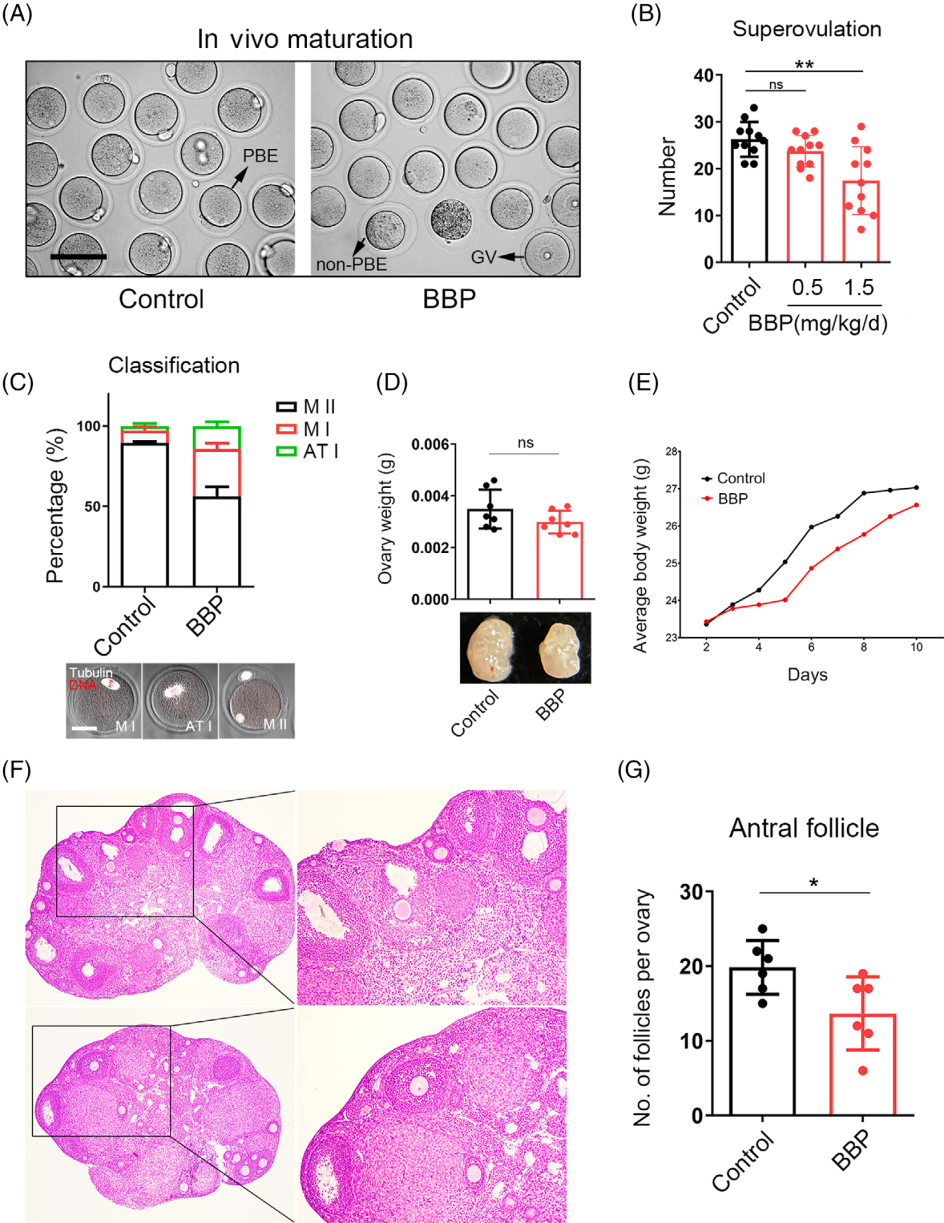
Benzyl butyl phthalate (BBP) exposure leads to a decrease in the number of oocyte and antral follicle, as well as the developmental potential of oocyte. (A) Representative images of the oocytes obtained from control and BBP‐exposed ovary after superovulation. (B) The number of oocytes obtained from BBP‐exposed (1.5 mg/kg body weight/day) group were significantly reduced comparing with control and BBP‐exposed (0.5 mg/kg body weight/day) groups. Results are expressed as mean ± SD; experiments were repeated at least three times (NS, not significant; *p < 0.05; **p < 0.01; *** *p* < 0.001). (C) Most oocytes obtained from BBP‐exposed group could only grow to MI stages, but most of control oocytes could reach to the MII phase. (D) Ovary weight was not changed after BBP exposure. Results are expressed as mean ± SD; experiments were repeated at least three times (NS, not significant; *p < 0.05; **p < 0.01; ***p < 0.001). (E) Average body weight was not changed after BBP exposure. (F) Pictures of control and BBP‐exposed ovary sections. (G) Number of antral follicles per ovary was significantly reduced in BBP‐exposed group than that in control. Results are expressed as mean ± SD; experiments were repeated at least three times (NS, not significant; *p < 0.05; **p < 0.01; ***p < 0.001).

Mice were exposed to BBP for 8 successive days and hormone injections were given on Days 8 and 10 for superovulation. In order to determine the optimal dose, various concentrations of BBP (0.5 and 1.5 mg/kg body weight/day) were administered. When the concentration of BBP increased to 1.5 mg/kg body weight/day, the number of oocytes obtained from each ovary and the in vivo maturation rate of oocytes were significantly reduced in BBP treated group after superovulation (Control: 26.3 ± 1.5, *n* = 11; BBP: 17.5 ± 1.3, *n* = 11, *p* < 0.01; Figure 1A,B). By fluorescence staining of spindle and chromosome, it was found that oocytes which did not develop to MII stage were mainly stagnated in MI and ATI after BBP exposure (Figure 1C). However, ovary weight and average body weight (Control: 0.00348 ± 0.0003, *n* = 7; BBP: 0.00298 ± 0.0004, *n* = 7, *p* = 0.1558; Figure 1D) were not different between each group (Figure 1E). The dose of 1.5 mg/kg body weight/day was used for our further study. As shown in Figure 1F, we discovered that compared with the control group, the number of antral follicles was reduced in the BBP‐exposed group (Control: 19.8 ± 1.4, *n* = 6; BBP: 13.7 ± 1.6, *n* = 6, *p* < 0.05; Figure 1G). Our results indicated that BBP exposure could lead to a severe decrease of oocyte amount and inhibit the development of oocytes and antral follicles.

### 3.2 BBP exposure affects mouse oocyte meiotic maturation

**FIGURE 2:**
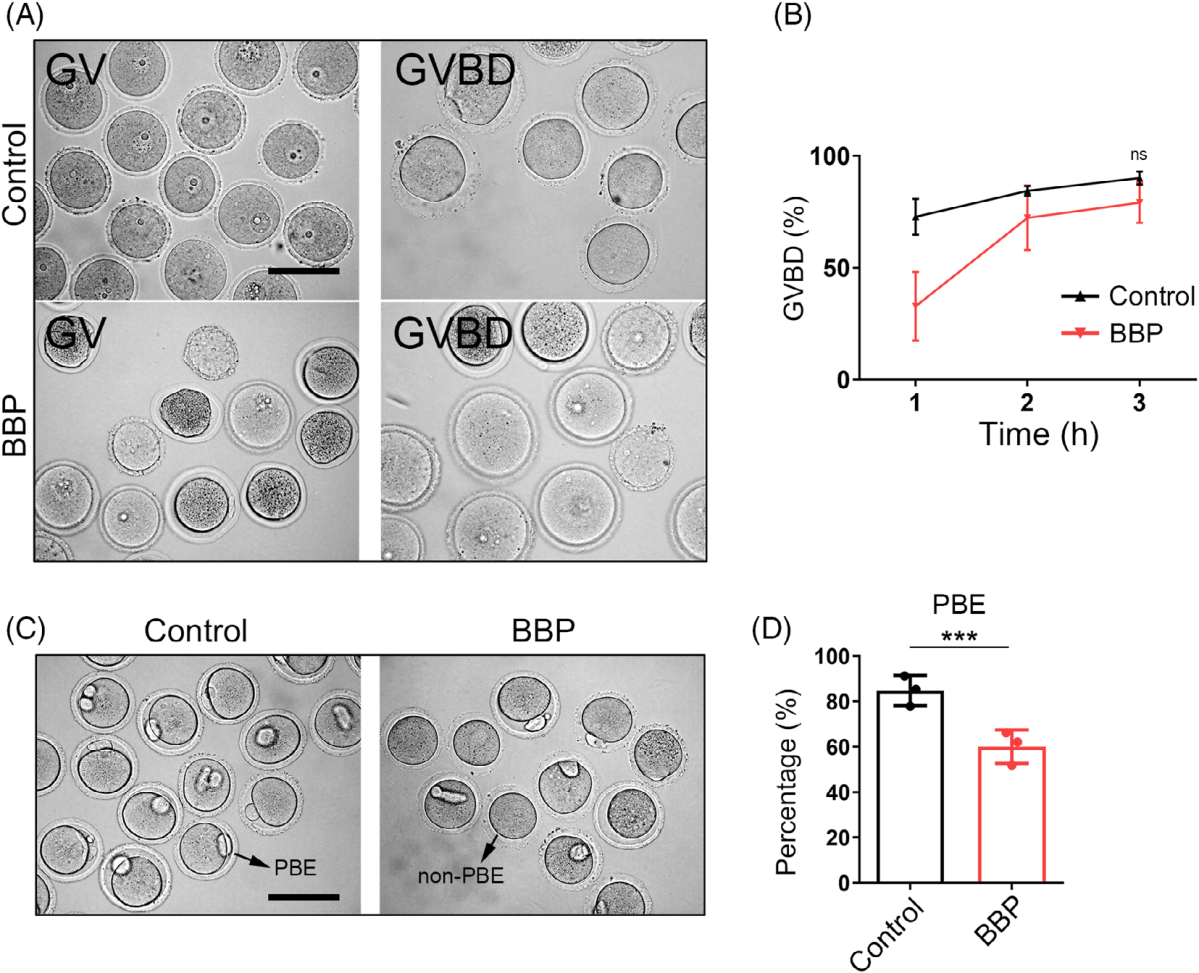
Benzyl butyl phthalate (BBP) exposure affects oocyte meiotic maturation. (A) Representative images of the germinal vesicles oocytes obtained from control and BBP‐exposed ovary, and images of the germinal vesicle breakdown (GVBD) oocytes cultured in vitro. (B) The GVBD rate of BBP‐exposed oocytes was not different from that of control oocytes after 3 h culture. Results are expressed as mean ± SD; experiments were repeated at least three times (NS, not significant; *p < 0.05; **p < 0.01; ***p < 0.001). (C) Representative images of oocytes in control and BBP‐exposed groups after 12 h culture. (D) The rate of polar body extrusion was significantly reduced in the BBP‐exposed oocytes comparing with that of the control oocytes. Results are expressed as mean ± SD; experiments were repeated at least three times (NS, not significant; *p < 0.05; **p < 0.01; ***p < 0.001).

Oocytes in GV stage were obtained from BBP‐exposed and control mice, and were cultured in vitro for subsequent analysis. GV oocytes in BBP treatment group were smaller in size and darker in cytoplasm obviously (Figure 2A). And, the GVBD rate of BBP exposure oocytes was not significantly different compared with the control group after 3 h culture (Figure 2B). As shown in Figure 2C, most of oocytes in control group succeeded to reach MII phase and extruded the polar body after 12 h culture. While in BBP‐exposed group, oocytes failed to accomplish the meiotic maturation, and cannot extrude the first polar body. When compared with the control group, the rate of polar body extrusion significantly reduced in the BBP‐exposed oocytes (Control: 84.8% ± 2.5%, *n* = 97; BBP: 60.07% ± 2.7%, *n* = 91, *p* < 0.001; Figure 2D). Therefore, BBP exposure has no effect on the meiotic resumption of mouse oocyte but can remarkably affect oocyte maturation.

### 3.3 BBP exposure affects the fertilization ability and early embryo development of mouse oocyte

**FIGURE 3:**
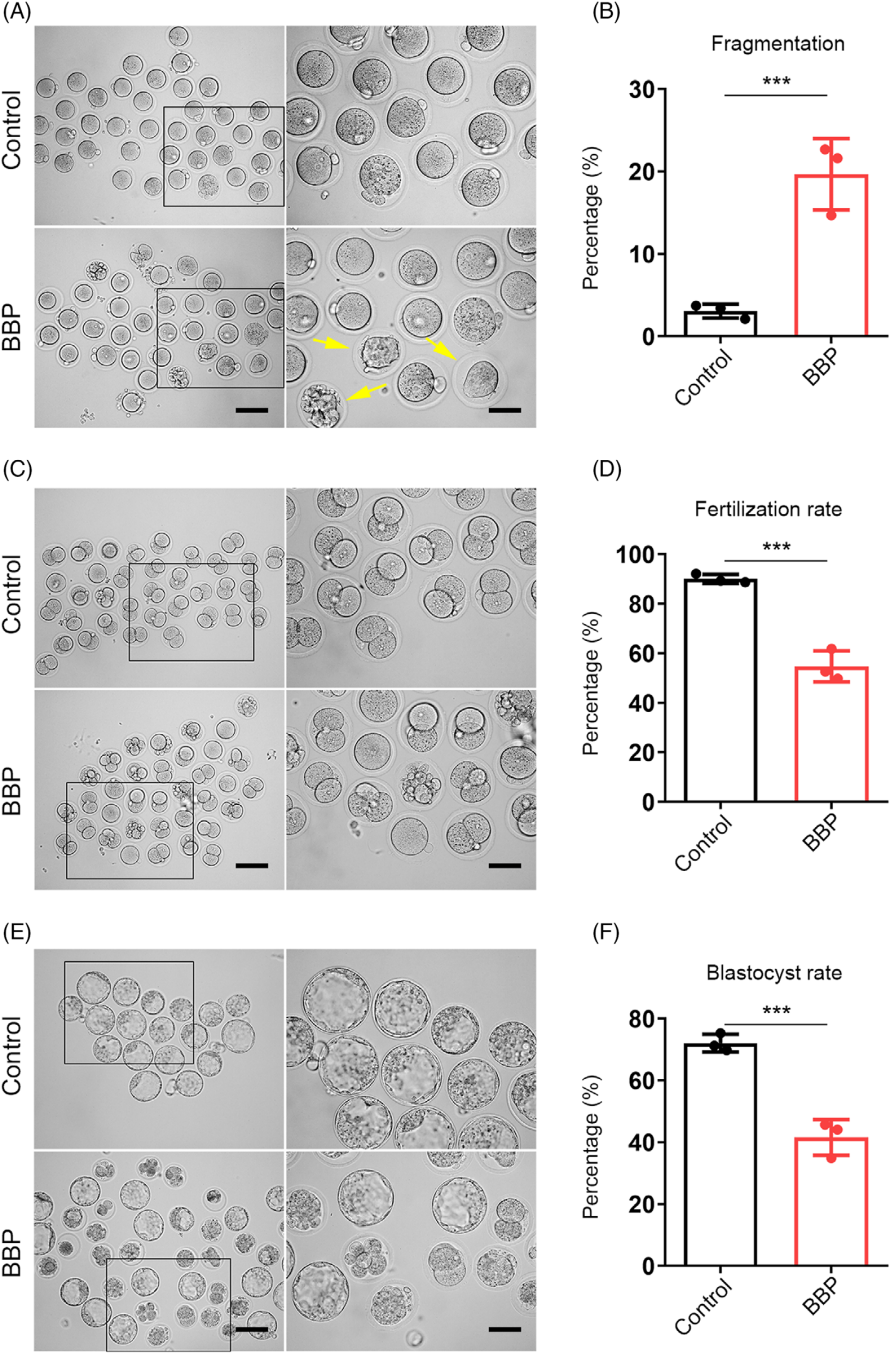
Benzyl butyl phthalate (BBP) exposure affects the fertilization ability and early embryo development of oocytes. (A) Representative images of fragmented MII oocytes obtained from control and BBP‐exposed ovary after superovulation. (B) BBP‐exposed group displayed an apparently increased fragmentation rate comparing with that of control oocytes. Results are expressed as mean ± SD; experiments were repeated at least three times (NS, not significant; *p < 0.05; **p < 0.01; ***p < 0.001). (C) Representative images of two‐cell embryos in control and BBP‐exposed groups after in vivo fertilization. (D) BBP‐exposed group had a significantly lower fertilization rate than control group. Results are expressed as mean ± SD; experiments were repeated at least three times (NS, not significant; *p < 0.05; **p < 0.01; ***p < 0.001). (E) Representative images of blastocyst in control and BBP‐exposed groups after further culture. (F) The rate of blastocyst formation in BBP‐exposed group was significantly declined comparing with that of the control. Results are expressed as mean ± SD; experiments were repeated at least three times (NS, not significant; *p < 0.05; **p < 0.01; ***p < 0.001).

We also found that BBP‐exposed oocytes displayed an apparently increased fragmentation rate comparing with that of control oocytes (Figure 3A). Statistical analysis also confirmed that (Control: 3.07% ± 2.6%, *n* = 110; BBP: 19.67% ± 2.9%, *n* = 119, *p* < 0.001; Figure 3B). We subsequently investigated that whether the fertilization capacity of oocytes was weakened by BBP exposure. In vivo fertilization experiment displayed that most of control oocytes were fertilized and developed into two‐cell embryos, while BBP‐exposed oocytes exhibited a significantly lower fertilization rate (Control: 90.03% ± 1.8%, *n* = 90; BBP: 54.73% ± 1.9%, *n* = 89, *p* < 0.001; Figure 3C,D). With further culture, the result showed that BBP exposure significantly reduced the blastocyst formation rate of fertilized oocytes (Figure 3E). Data analysis showed that the rate of blastocyst formation in BBP‐exposed group was significantly declined comparing with that of the control (Control: 72.1% ± 1.7%, *n* = 88; BBP: 41.57% ± 2.3%, *n* = 81, *p* < 0.001; Figure 3F). These results indicated that BBP also destroyed the fertilization ability of oocytes and inhibited the subsequent development of early embryos.

### 3.4 BBP exposure leads to spindle/chromosome disruption and decreased ac‐tubulin levels in mouse oocyte

**FIGURE 4:**
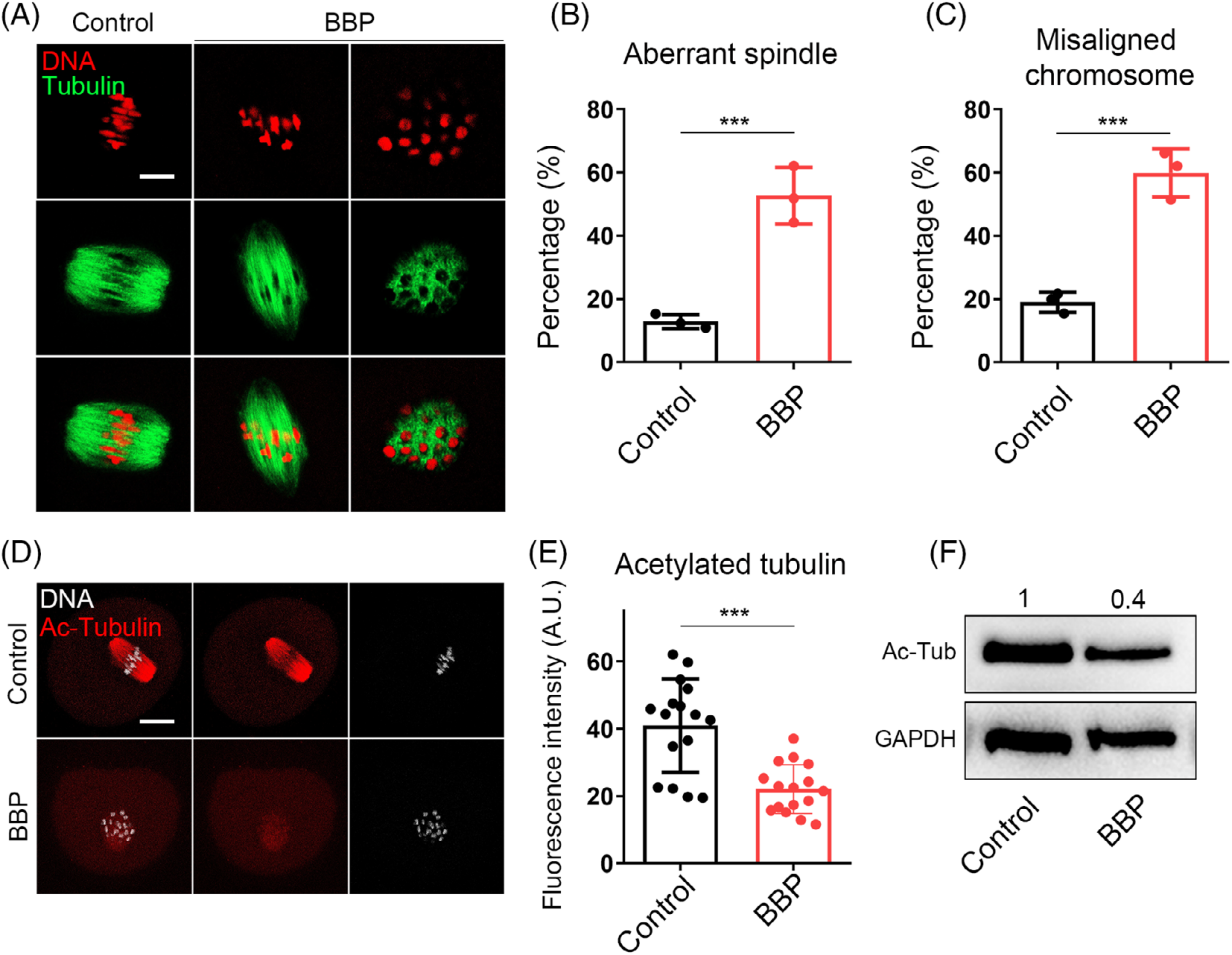
Benzyl butyl phthalate (BBP) exposure leads to spindle/chromosome disruption and decreased ac‐tubulin levels in mouse oocyte. (A) Representative images of spindle and chromosome in the MI oocyte from control and BBP‐exposed groups. Green, α‐tubulin; red, DNA. (B) The percentage of MI oocytes with aberrant spindle in BBP‐exposed group was significantly higher than that in the control group. Results are expressed as mean ± SD; experiments were repeated at least three times (NS, not significant; *p < 0.05; **p < 0.01; ***p < 0.001). (c) The percentage of MI oocytes with misaligned chromosome in BBP‐treated group was significantly higher than that in the control group. Results are expressed as mean ± SD; experiments were repeated at least three times (NS, not significant; *p < 0.05; **p < 0.01; ***p < 0.001). (D) Representative images of ac‐tubulin in the control and BBP‐exposed groups. Red, ac‐tubulin; grey, DNA. (E) The fluorescence intensity of ac‐tubulin in BBP‐exposed oocytes was much lower than that in the control oocytes. Results are expressed as mean ± SD; experiments were repeated at least three times (NS, not significant; *p < 0.05; **p < 0.01; ***p < 0.001). (F) Western blot result also displayed a declined level of ac‐tubulin. K‐M, kinetochore–microtubule.

We next detected the cytoskeleton morphology and dynamics in MI oocytes from each group. As shown in Figure 4A, most control oocytes displayed the classic spindle of shuttle shape and the chromosome were well aligned on the central plate. However, spindle assembly and chromosomes assignment were fearfully destroyed in BBP‐exposed oocytes. The percentage of oocytes with aberrant spindle and misaligned chromosome in BBP‐treated group was significantly higher than that in the control group (disorganized spindle: 12.8% ± 2.1%, *n* = 79, control vs. 52.7% ± 1.9%, *n* = 81, HS, *p* < 0.01; misaligned chromosome: 18.9% ± 1.9%, *n* = 79 control vs. 59.9% ± 1.3%, *n* = 81, HS, *p* < 0.001; Figure 4B,C). To point out the potential mechanism of BBP on spindle formation during mouse oocyte meiosis, we examined the tubulin acetylation level in both group, which was the marker of stable microtubules. We found that the fluorescence intensity of ac‐tubulin in BBP‐exposed oocytes was much lower than that in the control oocytes (Figure 4D). The statistics analysis also confirmed that (Control: 40.96 ± 2.1, *n* = 16; BBP: 22.08 ± 1.7, *n* = 16, *p* < 0.001; Figure 4E). Moreover, the western blot results came to the coincident conclusion (Figure 4F). This suggested that BBP disturbed the spindle organization and chromosome alignment through its effect on tubulin acetylation.

### 3.5 BBP exposure leads to defective K‐M attachment and aneuploidy in mouse oocyte

**FIGURE 5:**
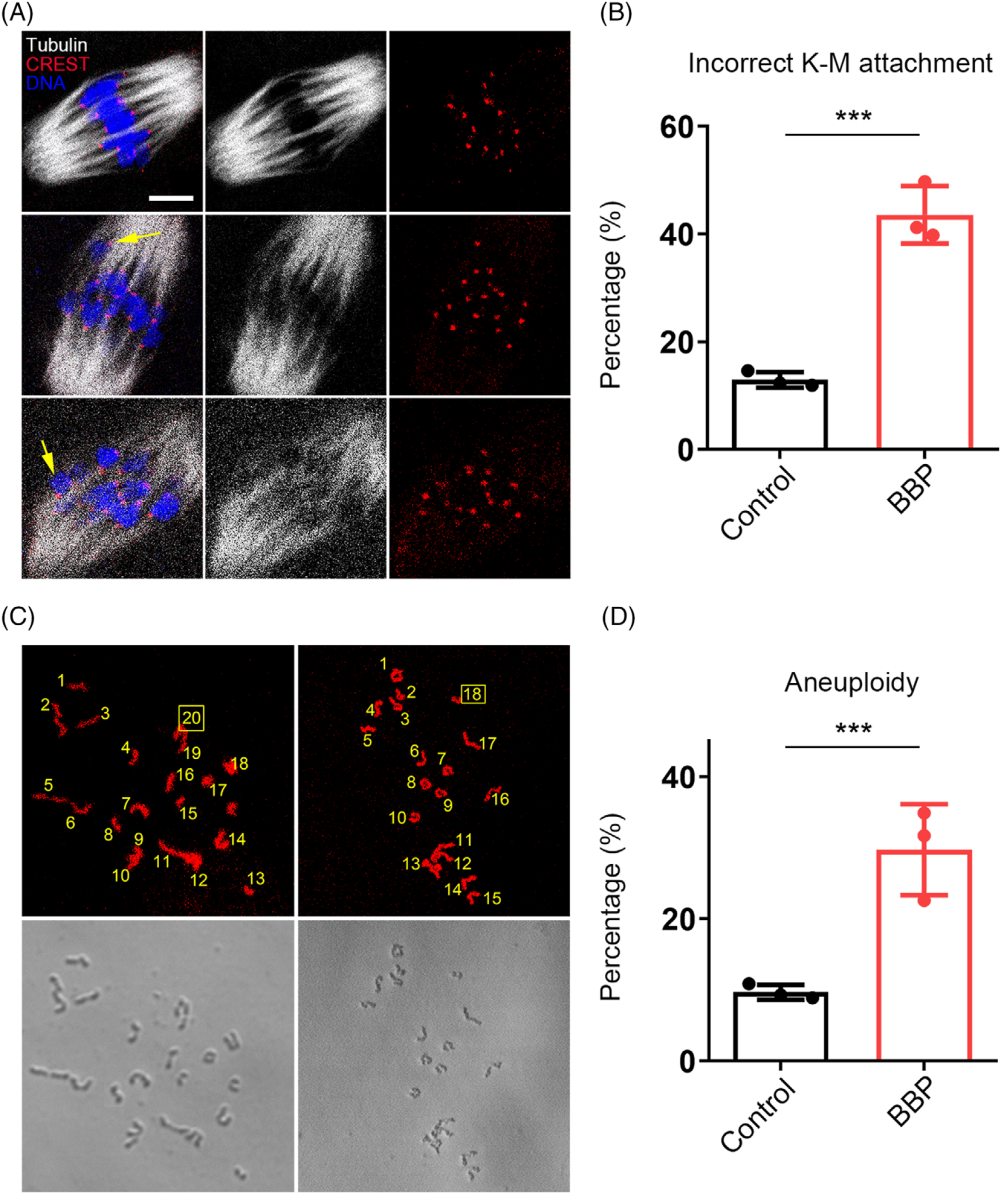
Benzyl butyl phthalate (BBP) exposure leads to defective kinetochore–microtubule (K‐M) attachment and aneuploidy in mouse oocyte. (A) Representative images of the morphology of K‐M attachments in MI oocytes from both control and BBP‐exposed group. Grey, α‐tubulin; red, CREST; blue, DNA. (B) The rate of incorrect K‐M attachment in BBP‐exposed group was significantly higher than that in control group. Results are expressed as mean ± SD; experiments were repeated at least three times (NS, not significant; *p < 0.05; **p < 0.01; ***p < 0.001). (C) Representative images of the number of chromosomes through chromosome spreading on MII oocytes in each group. Red, DNA. (D) Aneuploidy in BBP‐exposed group was prominently increased comparing with that in the control group. Results are expressed as mean ± SD; experiments were repeated at least three times (NS, not significant; *p < 0.05; **p < 0.01; ***p < 0.001).

Spindle disorganization and chromosome misalignment are usually associated with the failed attachment of K‐M. We then stained the oocytes of each group to detect the K‐M attachments morphology with CREST. MI oocytes from both control and BBP‐exposed group were chilled in 4°C for 10 min to induce the depolymerization of unstable microtubules. As shown in Figure 5A, most control oocytes displayed well‐attached kinetochores on the central plate. While in the BBP‐exposed oocytes, the K‐M attachments were defective and the chromosomes were scattered. As statistical analysis, the rate of incorrect K‐M attachment in BBP‐exposed group was significantly higher than that in control group (Control: 12.9% ± 2.5%, *n* = 27; BBP: 43.6% ± 2.1%, *n* = 31, *p* < 0.001; Figure 5B). The K‐M attachment failures and chromosomes separation errors would induce the generation of aneuploidy ovum. Through chromosome spreading on MII oocytes in each group, we found that aneuploidy from the BBP‐exposed group was prominently increased comparing with that in the control group (Control: 9.7% ± 1.6%, *n* = 29; BBP: 29.7% ± 1.9%, *n* = 30, *p* < 0.001; Figure 5C,D). These results indicated that BBP exposure caused meiotic defects through inducing failed K‐M attachment and eventual aneuploidy in mouse oocyte.

### 3.6 BBP exposure affects the dynamics of Actin and cortical granules in mouse oocyte

**FIGURE 6:**
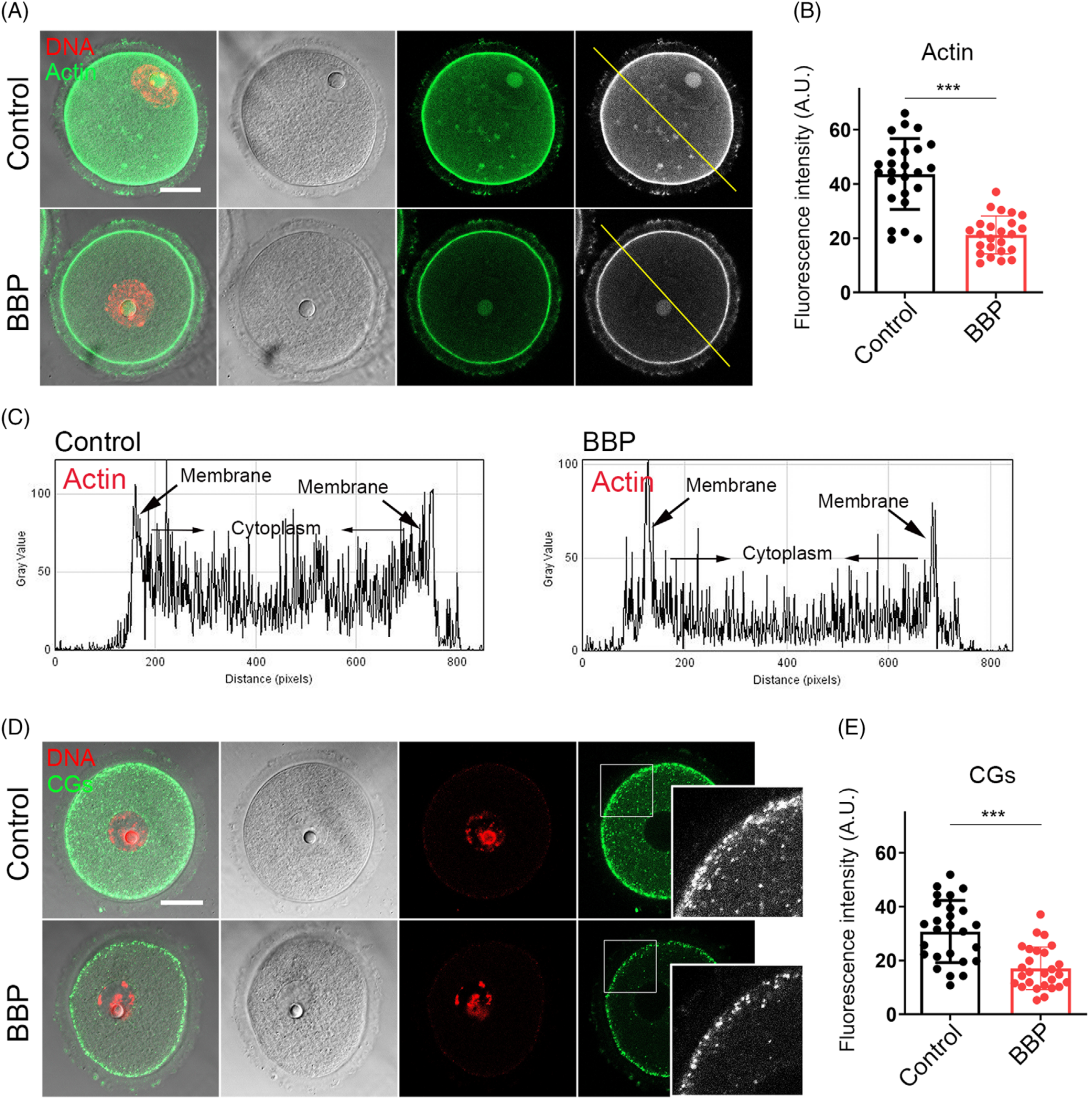
Benzyl butyl phthalate (BBP) exposure affects the dynamics of Actin and cortical granules. (A) Germinal vesicle (GV) oocytes from each group were stained by phalloidin, and representative images of actin signal in control and BBP‐exposed oocytes. Green, Actin; red, DNA. (B) The average fluorescence intensity of actin was significantly decreased in BBP‐exposed oocytes comparing with that in the control oocytes. Results are expressed as mean ± SD; experiments were repeated at least three times (NS, not significant; *p < 0.05; **p < 0.01; ***p < 0.001). (C) Both cytoplasmic actin and cortical actin showed lower fluorescence intensity after BBP exposure. (D) GV oocytes from each group were stained by lens culinary agglutinin‐FITC. The representative images of CGs signal in control and BBP‐exposed oocytes. Green, CGs; red, DNA. (E) The fluorescence intensity of cortically distributed CGs was significantly decreased in BBP‐exposed oocytes comparing with that in the control oocytes. Results are expressed as mean ± SD; experiments were repeated at least three times (NS, not significant; *p < 0.05; **p < 0.01; ***p < 0.001).

Given that actin filaments dynamics is also closely associated with mouse oocyte maturation, we subsequently detected the oocyte actin dynamics after BBP exposure. GV oocytes from each group were stained by phalloidin, a specific dye for cellular actin. As shown in Figure 6A, the signal intensity of actin was obviously declined after BBP exposure. As the statistics of fluorescence intensity, both cytoplasmic actin and cortical actin were remarkably decreased in BBP‐exposed group comparing with that in the control oocytes (Control: 43.6 ± 2.1, *n* = 25; BBP: 21.2 ± 1.9, *n* = 24, *p* < 0.001; Figure 6B,C). Cortical granules (CGs) are specific vesicles in mammalian oocyte and are located to the subcortex for polyspermy blocking. In general, the successful distribution of CGs is essential for oocyte cytoplasmic maturation. Using lens culinary agglutinin‐FITC staining, we assessed the distribution and dynamics of CGs after BBP exposure. In control oocytes, CGs distributed uniformly in the subcortex. But in BBP‐exposed oocytes, CGs lost the classic localization pattern and displayed a weaker signal (Figure 6D). The statistics of CGs fluorescence intensity also confirmed this (Control: 30.8 ± 2.6, *n* = 26; BBP: 17.1 ± 2.1, *n* = 27, *p* < 0.001; Figure 6E). These results suggested that BBP exposure destroyed actin assembly and CGs dynamic during mouse oocyte meiosis.

### 3.7 Transcriptome analysis for the global mRNA expression in the BBP‐exposed mouse oocyte

**FIGURE 7:**
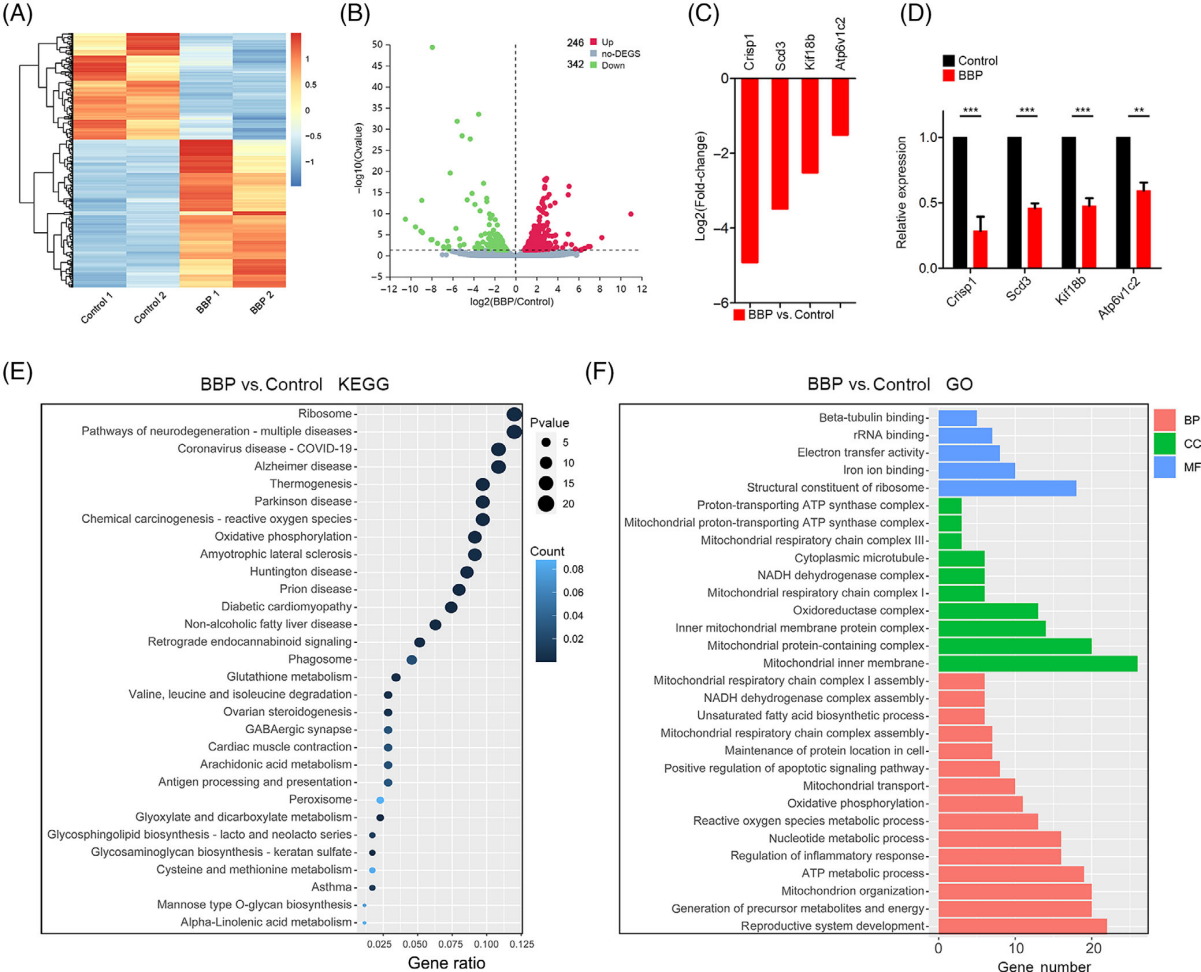
Transcriptome analysis for the global mRNA expression in the benzyl butyl phthalate (BBP)‐exposed oocytes. (A) Heatmap of differentially expressed genes (DEGs) in control and BBP‐exposed oocytes. (B) Volcano plot of DEGs in control and BBP‐exposed oocytes. (C) The expression of several randomly selected genes (*Crisp1*; *Scd3*; *Kif18b*; *Atp6v1c2*) was declined after BBP exposure. (D) The mRNA levels evaluated by quantitative real‐time PCR of the selected genes (*Crisp1*; *Scd3*; *Kif18b*; *Atp6v1c2*) in control and BBP‐exposed oocytes. Experiments were repeated at least three times (NS, not significant; *p < 0.05; **p < 0.01; ***p < 0.001). (E) Kyoto Encyclopedia of Genes and Genomes pathway enrichment analysis of DEGs in control and BBP‐exposed oocytes. (F) Gene ontology pathway enrichment analysis of DEGs in control and BBP‐exposed oocytes. Red represents biological processes, green represents cellular components, and blue represents molecular function.

To further illustrate the underlying mechanism of BBP toxicity on mouse oocyte maturation, we conducted single‐cell transcriptome analysis of both BBP‐exposed and control oocytes. When compared with the control oocytes, the heatmap and volcano plot results displayed that the BBP exposure oocytes transcriptome profile was significantly different. A total of 342 differentially expressed genes (DEGs) were down‐regulated and 246 DEGs were upregulated (Figure 7A,B). The expression of several randomly selected genes (*Crisp1*; *Scd3*; *Kif18b*; *Atp6v1c2*) was declined after BBP exposure, and the same result was verified by quantitative real‐time PCR (Figure 7C,D). KEGG results demonstrated that multiple differently expressed genes were enriched in the mitochondrial function‐associated pathway (Figure 7E). Moreover, GO analysis demonstrated that the mitochondrial transport, mitochondrial respiratory chain, tubulin and ATP metabolism‐related genes were mis‐expressed in BBP‐exposed oocytes (Figure 7F). Collectively, the pathways and biological processes are closely associated with mitochondrial function, which prompts us to investigate the change in mitochondria function after BBP exposure.

### 3.8 NMN recovers mitochondrial function and reduces oxidative stress in BBP‐exposed mouse oocyte

**FIGURE 8:**
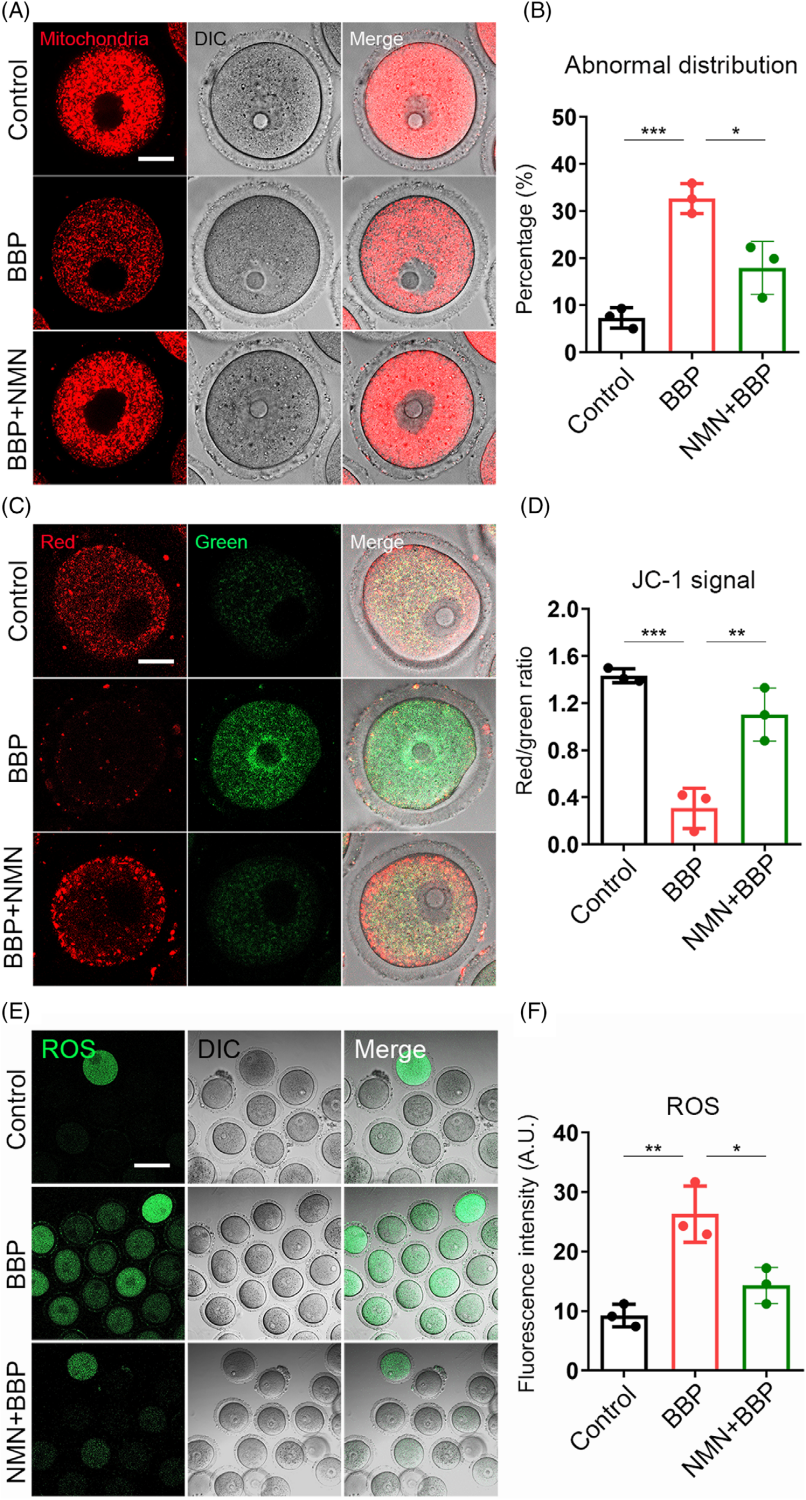
Nicotinamide mononucleotide (NMN) recovers mitochondrial function and reduces oxidative stress in benzyl butyl phthalate (BBP)‐exposed mouse oocyte. (A) Germinal vesicle (GV) oocytes from each group were stained by Mito Tracker, and representative images of mitochondrial in control, BBP‐exposed and NMN + BBP oocytes. Red, Mitochondria. (B) BBP‐exposed oocytes displayed a significantly higher rate of the mis‐localized mitochondria than that in control oocytes, and NMN‐supplemented rescued the abnormal rate. Results are expressed as mean ± SD; experiments were repeated at least three times (NS, not significant; *p < 0.05; **p < 0.01; ***p < 0.001). (C) GV oocytes from each group were stained by JC‐1 to assess the mitochondrial membrane potential. The representative images of JC‐1 signal in control, BBP‐exposed and NMN + BBP oocytes. Red/green, JC‐1. (D) The ratio of red signal to green signal was significantly lower in BBP‐exposed oocytes than that in control oocytes, while NMN supplementation rescued this abnormality. (E) GV oocytes from each group were stained by dichlorofluorescein, and representative images of reactive oxygen species (ROS) signal in control, BBP‐exposed and NMN + BBP oocytes. Green, ROS. (F) BBP‐exposed oocytes appeared significantly higher ROS level than that in control oocytes, and NMN supplement recovered the ROS level after BBP exposure. Results are expressed as mean ± SD; experiments were repeated at least three times (NS, not significant; *p < 0.05; **p < 0.01; ***p < 0.001).

Next, the effect of BBP on mitochondrial function of mouse oocytes would be verified. First, the mitochondria distribution of oocyte in each group was detected by Mito Tracker staining. We found that mitochondrial in control oocytes homogeneously distributed around the chromosome periphery. While in BBP‐exposed oocytes, cytoplasmic mitochondrial lost the classical accumulation and presented a weaker signal. Moreover, NMN‐supplement recovered the disordered distribution of mitochondria after BBP exposure (Figure 8A). As statistical analysis, BBP‐exposed oocytes displayed a significantly higher rate of the mitochondria abnormal distribution than that in control oocytes, and NMN‐supplemented rescued the abnormal rate (Control: 7.3% ± 1.3%, *n* = 77; BBP: 32.7% ± 1.2%, *n* = 81, *p* < 0.001; NMN + BBP: 17.9% ± 1.7%, *n* = 82, *p* < 0.05; Figure 8B). We subsequently tested the mitochondrial membrane potential by JC‐1 staining, which promotes the ATP synthesis in mitochondrial. Mitochondria with high membrane potential showed red signal, whereas green signal represents mitochondria with low membrane potential (Figure 8C). We found that compared with the control oocytes, the red‐green signal ratio of BBP‐exposed oocytes was significantly lower, while NMN supplementation rescued this abnormality (Control: 1.4 ± 1.1, *n* = 69; BBP: 0.3 ± 2.1, *n* = 75, *p* < 0.001; NMN + BBP: 1.1 ± 1.9, *n* = 70, *p* < 0.01; Figure 8D). Mitochondrial dysfunction is the main reason for ROS accumulation and oxidative stress. Therefore, we performed dichlorofluorescein (DCFH) staining to detect the level of ROS in oocytes from each group (Figure 8E). Fluorescence intensity measurements showed that BBP‐exposed oocytes appeared significantly higher ROS level than that in control oocytes, and NMN supplement recovered the ROS level after BBP exposure (Control: 9.2 ± 1.0, *n* = 66; BBP: 26.3 ± 1.9, *n* = 70, *p* < 0.01; NMN + BBP: 14.3 ± 1.4, *n* = 71, *p* < 0.05; Figure 8F). Altogether, these data suggest that BBP exposure could induce mitochondrial dysfunction and oxidative stress in mouse oocyte, and this condition could be rescued by NMN supplementation.

### 3.9 NMN decreases DNA damage and early apoptosis induced by BBP exposure in mouse oocyte

**FIGURE 9:**
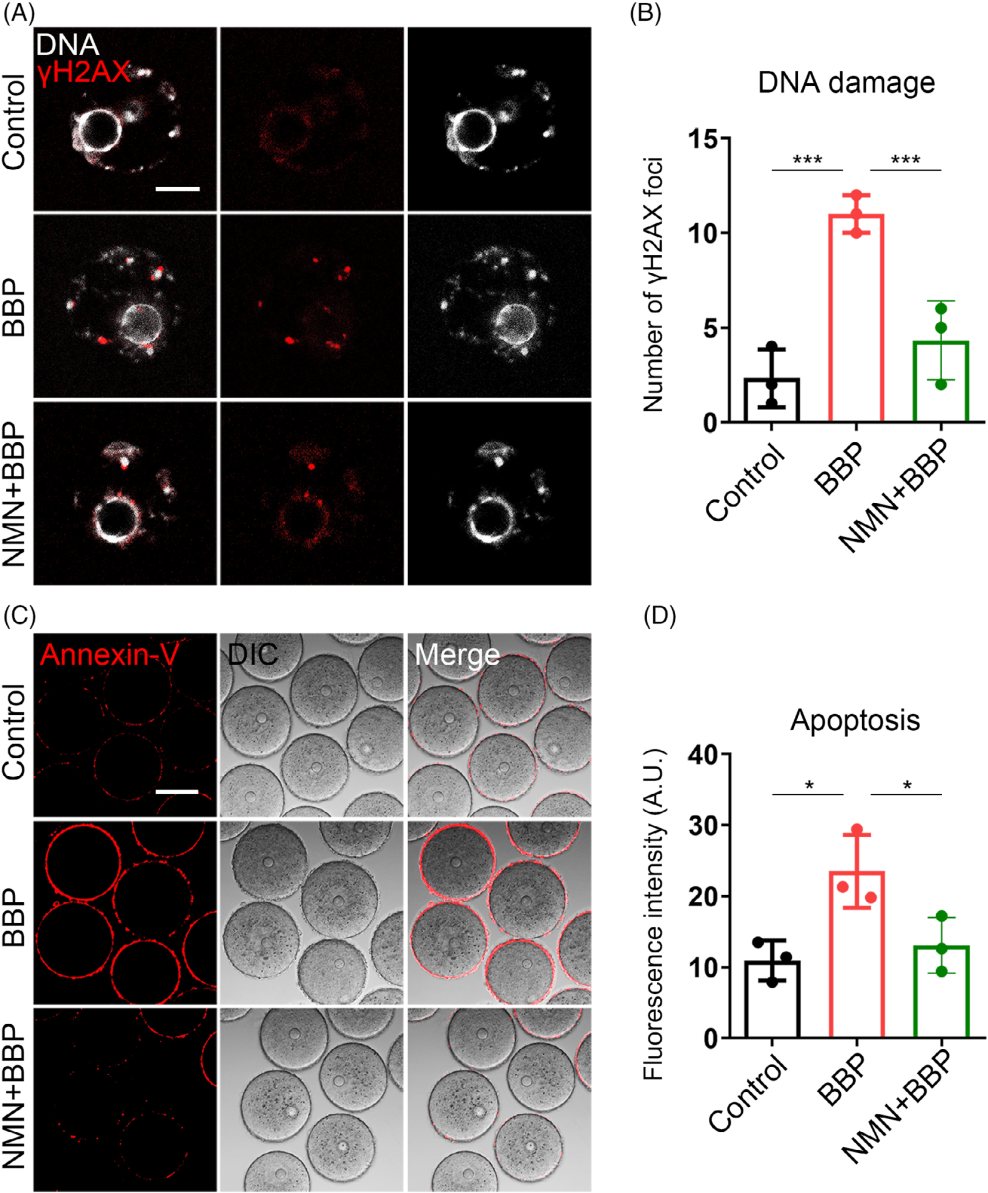
Nicotinamide mononucleotide (NMN) decreases DNA damage and early apoptosis induced by benzyl butyl phthalate (BBP) exposure in oocytes. (A) Germinal vesicle (GV) oocytes from each group were stained by γ‐H2A.X to assess the level of DNA damage, and representative images of γ‐H2A.X signal in control, BBP‐exposed and NMN + BBP oocytes. Red, γ‐H2A.X; grey, DNA. (B) The level of DNA damage was obviously increased after BBP exposure and NMN supplementation effectively suppressed this condition in oocyte. Results are expressed as mean ± SD; experiments were repeated at least three times (NS, not significant; *p < 0.05; **p < 0.01; ***p < 0.001). (C) GV oocytes from each group were stained by Annexin‐V to assess the level of early apoptosis, and representative images of Annexin‐V signal in control, BBP‐exposed and NMN + BBP oocytes. Red, Annexin‐V. (D) The level of early apoptosis was significantly increased after BBP exposure and NMN supplement effectively recovered it in oocyte. Results are expressed as mean ± SD; experiments were repeated at least three times (NS, not significant; *p < 0.05; **p < 0.01; ***p < 0.001).

We subsequently assessed the levels of DNA damage and early apoptosis by γ‐H2A.X staining and Annexin‐V staining, in oocytes from each group, respectively, considering that excessive ROS usually leads to DNA damage accumulation and induces early apoptosis. As we expected, the levels of DNA damage and early apoptosis were obviously increased after BBP exposure, and NMN supplementation effectively suppressed this condition in mouse oocyte (Figure 9A,C). Meanwhile, statistical analysis confirmed the same result (DNA damage: Control: 2.3 ± 1.6, *n* = 70; BBP: 11.0 ± 1.4, *n* = 79, *p* < 0.001; NMN + BBP: 4.3 ± 2.1, *n* = 81, *p* < 0.001; Figure 9B. Apoptosis: Control: 10.9 ± 1.5, *n* = 81; BBP: 23.5 ± 1.9, *n* = 79, *p* < 0.05; NMN + BBP: 13.1 ± 1.7, *n* = 82, *p* < 0.05; Figure 9D). The results suggest that BBP exposure disrupted mouse oocyte maturation through inducing DNA damage and early apoptosis, and the effect could be rescued by NMN supplement.

## 4 DISCUSSION

It is well known that endocrine disruptors, such as bisphenol A, 4‐chloro‐3‐methyl phenol, di(2‐ethylhexyl) phthalate and BBP have adverse effects on meiotic maturation, cumulus expansion and hormone synthesis, such as follicle‐stimulating hormone, in most mammals.^18^ BBP is a widely used chemical material that is harmful for human health because of its estrogenic activity and pathogenicity. The present study investigated the effects of BBP on mouse oocyte maturation and early embryo development.

This study showed that the BBP reduced the total number of antral follicles in mice. Additionally, BBP exposure decreased the developmental potential of mouse oocytes. Examination of the influence of BBP exposure on GVBD and polar body extrusion of oocytes found that BBP induced MI arrest. In addition, this study revealed that BBP exposure induced the failure of oocyte fertilization and early embryo cleavage.

The cytoskeleton mainly consists of microtubules and actin microfilaments, which are critical for the regulation of spindle‐mediated oocyte maturation.^19^ This study showed that BBP severely damaged spindle morphology and chromosome arrangement, as well as disrupted the actin and CGs dynamics in mouse oocytes. Mycotoxins, such as HT‐2 and zearalenone, can also cause meiotic defects by disabling the function of the cytoskeleton in mammalian oocytes.^20^, ^21^ Nonylphenol was reported to disrupt actin distribution and spindle assembly, thereby causing the failure of mouse oocyte maturation.^22^ Spindle abnormalities are generally associated with aberrant interactions between kinetochores and microtubules.^23^ Our results showed that K‐M attachment was severely disrupted in BBP‐exposed oocytes, likely leading to the disrupted chromosome separation and increased aneuploidy. These BBP‐induced actions are similar to those reported for bisphenol‐A, a pollutant in plastics that affects cytoskeleton organization and K‐M reactions in oocytes.^24^, ^25^ The current investigation of the reason for spindle disorganization found that BBP‐exposed oocytes had decreased levels of acetylated α‐tubulin, which is a marker of unstable microtubules in newly formed spindles.^26^, ^27^, ^28^ We propose that BBP‐induced changes to acetylation‐related microtubule stability may impact spindle assembly and K‐M interactions in mouse oocytes.

The present study further explored the underlying mechanism of BBP actions on mouse oocyte maturation and early embryo development. Transcriptome bioinformatics analysis revealed that BBP exposure altered the expression of genes associated with mitochondrial transport, respiratory chain and ATP metabolism in mouse oocytes, indicating abnormal mitochondrial function. Mitochondria are crucial for energy supplying and metabolites in various cell types.^29^ In oocytes, mitochondrial dysfunction may induce abnormal ATP synthesis and spindle organization, and it provides a likely reason for defective fertilization and embryo aneuploidy induced by BBP.^30^, ^31^, ^32^ In addition, BBP exposure disrupted mitochondrial distribution and membrane potential in mouse oocytes.^33^ Mitochondrial dysfunction generally results in ROS accumulation and oxidative stress.^29^, ^34^, ^35^ The current results showed that BBP exposure elevated the levels of ROS and contributed to strong oxidative stress in the oocyte cytoplasm.

NMN supplementation may effectively protect oocyte quality against environmental pollutant toxicity.^36^ Recently, NMN was reported to maintain oocyte quality and fertilization ability through the recovery of nuclear and cytoplasmic maturation in mice.^37^ This study found that NMN maintained mitochondrial function and reduced oxidative stress in BBP‐exposed oocytes. Mitochondrial dysfunction and excessive ROS can cause early apoptosis and DNA damage.^38^, ^39^, ^40^ Exposure to 2,2′4,4′‐tetrabromodiphenyl ether, a common homologue of polybrominated diphenyl ethers, damaged mouse oocyte quality via ROS accumulation and apoptosis.^41^ The current results showed that BBP exposure caused DNA damage and early apoptosis, detected by DNA damage marker γ‐H2AX and annexin‐V staining, respectively, in mouse oocytes. NMN supplementation effectively reduced the effects of BBP on DNA damage and apoptosis, consistent with NMN protecting mitochondrial function, and thus suppressing ROS accumulation, inhibiting DNA damage and reducing apoptosis of senescence oocytes.^17^ Overall, these findings suggest that oxidative stress from mitochondrial dysfunction may be the major pathway for BBP toxicity in oocytes, and NMN efficiently suppresses DNA damage and early apoptosis induced by BBP exposure.

## 5 CONCLUSION

To sum up, the current experiments demonstrated that BBP exposure induced mitochondrial dysfunction, causing ROS accumulation and thereby leading to oxidative stress‐mediated DNA damage and apoptosis, which ultimately resulted in meiotic defects in oocytes and fertilization failure in mice. Moreover, this study found that NMN administration reduced BBP toxicity and improved oocyte quality.

## AUTHOR CONTRIBUTIONS

Yan Chen and Yi Jiang conceived and designed the research; Yi Jiang, Di Wang, Rong Cheng, Yanan Pu, and Yangyang Jiao performed the research and acquired the data, Yi Jiang and Yan Chen analysed and interpreted the data. All authors were involved in drafting and revising the article.

## CONFLICT OF INTEREST STATEMENT

The authors declare no conflicts of interest.

## Data Availability

All data generated or analyzed during this study are included in this published article. All the data provided in this study are deposited in the repository(https://www.ncbi.nlm.nih.gov/), and the accession number (GSE215872).
